# Nonlinear association between provincial implementation intensity of county-level medical communities and patient flow patterns

**DOI:** 10.3389/fpubh.2026.1966297

**Published:** 2026-10-26

**Authors:** Ting Shi, Lan Yang, Yuehong Zhang

**Affiliations:** 1 School of Finance, Lanzhou University of Finance and Economics, Lanzhou, China; 2 School of Public Administration, Gansu University of Political Science and Law, Lanzhou, China

**Keywords:** integrated care, patient flow directions, tiered healthcare system, tightly integrated county-level medical communities, U-shaped relationship

## Abstract

**Introduction:**

As a key initiative of China’s healthcare system reform, tightly integrated county-level medical communities (CCMCs) are designed to optimize the allocation of high-quality medical resources and advance the tiered healthcare delivery system. However, evidence on their policy effects remains mixed, and most studies treat CCMC construction as a binary intervention while overlooking continuous variations in implementation intensity. Meanwhile, the international literature on integrated care and referral governance focuses largely on established universal health systems, with limited attention to how progressive reforms unfold nonlinearly in developing countries facing resource constraints and patient outflows. This study addresses two unresolved questions: does intensifying CCMC construction continuously promote local patient retention, or does it initially generate patient diversion toward county-level hospitals at the expense of grassroots resources?.

**Methods:**

Departing from prior work that relies on dummy variables, we construct a multidimensional CCMC Construction Intensity Index (CII) covering policy coverage, organizational integration, and institutional safeguards. Using provincial panel data from 2014 to 2024 and a two-way fixed-effects model, we identify the causal relationship between CII and patient flow patterns.

**Results:**

We observe a U-shaped association between CII and the relative scale of in-county visits, though this association is attenuated under more demanding trend controls and the estimated inflection point shows some sensitivity to sample composition. At lower levels of construction intensity, patient diversion toward county hospitals accompanies the resource integration process; once intensity crosses the threshold, substantive integration and long-term mechanisms are gradually unleashed, and patient retention within counties begins to dominate. This pattern is mirrored by an inverted U-shaped trend in out-of-county care-seeking. Heterogeneity analysis shows that these patterns are more pronounced in western regions and in areas with a higher density of private hospitals; within grassroots facilities, benefits accrue primarily to township health centers, while community stations and village clinics lag behind.

**Discussion:**

Theoretically, this study reveals that CCMC development in China follows a threshold-dependent trajectory, contributing Chinese evidence on the nonlinear evolution of referral dynamics to the global integrated care literature and helping reconcile mixed prior findings. From a policy perspective, the findings offer empirical support for calibrating implementation intensity to local contexts and for prioritizing township health centers as the main recipients of resource devolution, so as to mitigate early-stage patient diversion and support the gradual advancement of the tiered healthcare system.

## 1 Introduction

For a long time, the structural contradiction characterized by “overcrowded tertiary hospitals and underutilized primary care facilities” has plagued China’s healthcare system. As Yu Xiao et al. (1) point out, in recent years, the scale of large public hospitals in China has continued to expand at an unprecedentedly rapid pace; while hospitals have introduced large amounts of advanced equipment, this expansion has exacerbated the loss of qualified healthcare personnel from primary settings, further worsening the shortage of grassroots professionals. Meanwhile, due to the lack of a strong trust foundation for initial visits at the primary level and a smooth two-way referral mechanism, residents routinely bypass primary care and go directly to large tertiary hospitals even for common and frequently occurring conditions. This leaves large hospitals overwhelmed and physicians overworked, while high-quality primary care resources remain underutilized. As high-quality medical resources continue to concentrate in upper-level hospitals, bottlenecks persist in primary service capacity, and patients bear a heavier burden of cross-region medical expenses. As a result, the orderly care-seeking pattern envisioned by the tiered healthcare delivery system has yet to materialize effectively (2–4). To guide residents toward nearby care and optimize the referral order, the government has introduced multiple measures—such as raising medical insurance reimbursement rates for primary care and increasing financial investment in primary health—to strengthen primary service capacity. However, relying solely on economic incentives is unlikely to fundamentally alter residents’ preference for seeking care at higher-level facilities (5–7).

Against this backdrop, integrating county-level medical resources and advancing the construction of tightly integrated county-level medical communities (CCMCs) have become key institutional instruments for addressing the imbalance in medical resource allocation and reshaping patient flow patterns at the county level (8). Since the General Office of the State Council officially launched CCMC construction in 2017, the initiative has been rolled out nationwide with continuously expanding coverage. By the end of 2025, a total of 2,199 counties across China had established and put into routine operation tightly integrated county-level medical communities, integrating and covering 33,000 grassroots medical institutions in total (9, 10).

Existing literature has conducted extensive discussions on CCMC reform. Most studies confirm that the integrated CCMC model can promote the devolution of high-quality county-level medical resources and optimize the internal division of labor across county facilities, showing promise in strengthening primary care supply and reducing residents’ cross-region medical burdens, which lays a solid foundation for the present study. Nevertheless, existing empirical research still has three notable limitations. First, study samples are mostly limited to single pilot counties or short-term micro-survey data, lacking nationwide macro-panel evidence spanning a decade, which constrains the generalizability of findings. Second, econometric identification predominantly relies on binary dummy variables indicating whether a CCMC has been established, which cannot adequately capture the progressive implementation process characterized by uneven construction intensity across regions. Third, few studies have examined the nonlinear dynamic effects of the policy. They overlook the early-stage dual diversion of patients and healthcare personnel driven by county-level hospitals, as well as the county-level patient retention effects that emerge once mature profit-sharing mechanisms are in place. Furthermore, discussions on the inflection points and heterogeneity boundaries between patient outflow and patient return remain insufficient, leaving related theoretical propositions in need of more systematic and rigorous empirical testing.

Motivated by these practical and theoretical gaps, this paper uses provincial panel data from 2014 to 2024 to construct a multidimensional CCMC Construction Intensity Index (CII) covering three dimensions—policy coverage, organizational integration, and institutional safeguards. We employ a two-way fixed-effects model to examine the nonlinear association between CCMC implementation intensity and patient flow directions, and to explore its heterogeneity patterns. Relative to existing research, the marginal contributions of this study are threefold. First, in terms of research perspective, we move beyond the rigid binary dummy-variable setup common in policy evaluation and instead use a continuous intensity index to capture the gradual and differentiated nature of CCMC implementation, which more closely reflects how the policy unfolds at the local level. Second, in terms of data, we conduct a systematic assessment using a long-period national provincial panel, addressing the limitation of prior studies that rely on local pilots with limited representativeness. This provides more generalizable evidence on CCMC reform outcomes. Third, in terms of findings and theoretical dialogue, we identify a U-shaped association between CII and patient flow directions, though this nonlinear association is attenuated under more stringent trend controls. The results suggest a phased evolutionary pattern of “initial patient diversion followed by later retention,” and contribute Chinese empirical evidence on threshold-dependent dynamics to the international integrated care literature, helping scholars understand the phased logic of health service integration reforms under resource constraints. More broadly, constructing the CII and examining its nonlinear relationship with patient flow enriches research on macro-policy-driven institutional innovation (11) and offers generalizable evidence for evaluating Chinese-style institutional reforms.

The remainder of the paper is organized as follows. Section 2 provides the institutional background, literature review, and theoretical hypotheses. Section 3 describes the research design, including data sources, variable definitions, and econometric model specification. Section 4 presents the empirical results, covering baseline regressions, province-specific trend controls, robustness checks, sensitivity analyses, and heterogeneity analyses. Section 5 offers further discussion on the differential effects of CCMC construction across types of grassroots medical institutions. Section 6 concludes with policy recommendations.

## 2 Institutional background, literature review, and theoretical hypotheses

### 2.1 Institutional background

The tiered healthcare delivery system is a core institutional arrangement in China’s deepening healthcare reform. Its objective is to establish a collaborative division-of-labor mechanism featuring “initial diagnosis at the primary level, two-way referrals, separate management of acute and chronic conditions, and coordination across care levels,” thereby resolving the structural contradiction of “overcrowded tertiary hospitals and weak primary care capacity” and optimizing the efficiency of medical resource allocation.

In 2015, the General Office of the State Council issued the *Guiding Opinions on Promoting the Construction of a Tiered Healthcare Delivery System* (Guo Ban Fa [2015] No. 70), which explicitly identified “a significant increase in the share of outpatient visits at primary healthcare institutions” as a core quantitative evaluation indicator for the tiered healthcare system, laying the institutional foundation for subsequent reforms.

As the core operational vehicle of the tiered healthcare system at the county level, tightly integrated county-level medical communities (CCMCs) serve as a key platform for promoting the devolution of high-quality medical resources, strengthening primary care capacity, and building an integrated healthcare service system. Since the General Office of the State Council issued the *Guiding Opinions on Promoting the Construction and Development of Medical Alliances* in 2017 (Guo Ban Fa [2017] No. 32)—which called for a three-tier county network with “county hospitals as the lead, township health centers as hubs, and village clinics as the foundation”—CCMC development has evolved through successive stages: pilot initiation, criteria setting, comprehensive rollout, and standardized development, with policy ambition and implementation intensity steadily deepened (see Table 1).

**Table 1:** Major policy documents on compact county-level medical community construction.

Time	Document	Core Content
April 2017	*Guiding Opinions on Promoting the Construction and Development of Medical Alliances*	Mandated medical communities as the main model for county-level reform, and promoted the devolution of high-quality medical resources to grassroots facilities
May 2019	*Notice on Promoting the Construction of Tightly Integrated County-Level Medical Communities*	Launched the pilot program in Shanxi and Zhejiang provinces, covering 567 counties
August 2020	*Evaluation Criteria and Monitoring Indicator System for Tightly Integrated County Medical Communities (Trial)*	For the first time defined the four pillars of integration: responsibility, management, service, and benefit sharing
December 2023	*Guiding Opinions on Comprehensively Promoting Tightly Integrated County Medical Community Construction*	Marked the shift from pilots to nationwide rollout; clarified global budget payment and surplus retention mechanisms for medical insurance funds
October 2024	*Monitoring Indicator System for Tightly Integrated County Medical Communities (2024 Edition)*	Established 14 indicators across 5 dimensions, providing a quantitative monitoring framework
February 2025	*Guidelines on Informatization Functions for Tightly Integrated County Medical Communities*	Standardized 36 informatization functions across 5 domains
April 2026	*Several Measures for Accelerating the Construction of a Tiered Healthcare Delivery System*	Promoted integrated management; required each township health center to have at least one attending physician on site year-round

From the perspective of institutional operation logic, tightly integrated county-level medical communities (CCMCs) break down administrative barriers across county, township, and village institutions, thereby reconstructing regional two-way referral and coordination mechanisms. On the one hand, by promoting the devolution of high-quality medical resources and strengthening primary care capacity, CCMCs facilitate first-contact care at the primary level and smooth downward referrals, aiming to keep common and frequently occurring conditions at the grassroots level. On the other hand, by standardizing separate management of acute and chronic conditions and establishing fast-track referral pathways for upward transfers, CCMCs ensure that complex and severe cases are appropriately referred to county hospitals.

Although constrained by the granularity of provincial macro-panel data—making it impossible to directly observe individual-level referral trajectories—the “relative share of in-county visits” serves as a macro aggregate indicator that captures the combined effects of various upward and downward referral behaviors and the overall performance of the tiered healthcare system.

However, despite the continuous refinement of top-level institutional design, the tiered healthcare system still faces practical tensions in implementation. According to the National Health Commission, although primary care institutions nationwide now handle 52.6% of all outpatient visits (43), the share of hospital outpatient visits continues to rise as patients continue to concentrate in upper-level facilities. By contrast, within comprehensively promoted, tightly integrated CCMCs, the share of primary-level visits has exceeded 63%, significantly above the national average.

The coexistence of these structural contradictions and localized achievements suggests that the success of the tiered healthcare system depends not only on top-down institutional guidance but, more importantly, on the substantive deepening of CCMC integration. Driven by this alignment between policy goals and on-the-ground practice, CCMC development has entered a phase of rapid nationwide rollout. According to official NHC releases, by the end of 2025, a total of 2,199 counties (including county-level cities and agricultural districts) had established and were operating tightly integrated CCMCs, integrating 33,000 grassroots medical institutions. In addition, over 1,100 county-level centers for disease control and 1,600 county-level maternal and child health institutions have been incorporated into these networks, signaling that the county-level health system is accelerating toward greater integration and service standardization.

### 2.2 Literature review

This study systematically reviews research on healthcare service integration in China and internationally. Internationally, the World Health Organization (WHO) first proposed the concept of “integrated health services” in 1966 (12). Building on this, following the prospective payment system reforms of the 1980s, the United States pioneered a property-rights-based vertical integration model, incorporating nursing homes and primary clinics through mergers, acquisitions, and equity holding, with core operational features of “unified asset management, bundled payment, and shared surpluses.” (13–15).

The academic community remains divided on the effects of this model. On the negative side, property-rights integration may directly raise medical prices and increase medical insurance fund spending, an effect that is especially pronounced in less competitive markets (16–19); under this model, specialty hospitals exhibit cream-skimming behavior, preferentially attracting higher-margin patients (20); studies by Baker et al. (21, 22) and Cutler et al. (23) find that improvements in readmission rates and overall patient health have fallen short of expectations. On the positive side, the model can improve internal referral efficiency, promote the diversion of discharged patients to primary care, and achieve tiered resource utilization (24); unified management of personnel, finance, and materials streamlines service processes, shortening length of stay and reducing unnecessary visits (24, 25); furthermore, Carlin et al. (26, 27) and Solberg et al. (28) find that integration helps standardize referral pathways and strengthens basic services such as chronic disease follow-up and health screening.

Unlike US-style property-rights integration, the UK and Germany rely on universal health insurance systems to build models centered on service-process coordination—Germany strengthens community-level first-contact care through disease management programs, while the UK coordinates regional resources through integrated care systems, both emphasizing continuity of care across the patient journey (29, 30).

Research in China revolves around two main threads: urban medical alliances and county-level medical communities (CCMCs). Regarding urban medical alliances, studies affirm their positive roles in reducing operational costs and promoting two-way referrals (31, 32), though some scholars note that lead hospitals siphon patients away from grassroots facilities, so that tiered healthcare goals remain unmet (33); moreover, given substantial differences in organizational structure and lead-hospital tier between urban alliances and county CCMCs, findings from the former cannot be directly generalized to the latter (34).

Regarding CCMCs, Zhu Fengmei et al. (35) use large-scale insurance claims data to show that CCMCs help retain patients at the primary level and reduce chronic disease spending burdens; Gu Xin (36) points out that China’s CCMCs differ markedly in governance logic from both US property-rights integration and administratively organized urban alliances; other studies identify institutional constraints such as incomplete benefit-sharing mechanisms and shortages of grassroots healthcare personnel, which limit policy effectiveness (37, 38). Although a few studies indirectly suggest possible phased differences in CCMC effects, none has tested this possibility directly.

From a global perspective, within the integrated care and health system reform literature, patient referral and primary-level patient retention have long been central themes in international health policy research. The United States and the United Kingdom, relying on either property-rights vertical integration or universal insurance systems, have developed relatively mature referral and patient retention governance frameworks through mandatory GP gatekeeping, Diagnosis-Related Groups (DRG) prospective payment, and seamless information sharing. However, much of the international integrated care literature focuses on well-developed healthcare markets or mature universal health systems, with little attention to how incremental policy reform shapes the nonlinear evolution of referral mechanisms in developing or transitional economies where primary care networks face resource constraints and patient outflows. Although domestic Chinese research has accumulated rich on-the-ground evidence on urban alliances and CCMCs, most studies are confined to local pilots or binary dummy-variable specifications, lacking a systematic framework that situates China’s experience within the global integrated care and referral-retention literature. Building on these prior studies, this paper uses nationwide provincial panel data to conduct multidimensional empirical tests of CCMC implementation performance.

### 2.3 Theoretical mechanisms and research hypotheses

The impact of CCMC construction on patient flow directions is essentially a process in which supply-side structural reform reshapes the organizational models and operational mechanisms of county-level healthcare services, thereby altering residents’ care-seeking behavior. Accordingly, this study proposes the following theoretical hypotheses.

According to Andersen’s behavioral model of health services use, residents’ care-seeking behavior is shaped by three dimensions: predisposing characteristics, enabling resources, and need factors. The core logic of CCMC policy intervention is to reduce the need for cross-regional care-seeking by strengthening the enabling resources of county-level healthcare, including accessibility and supply capacity. CCMCs establish a “four-in-one” framework of responsibility, management, service, and benefit integration, achieving unified management of personnel, finance, and materials across county, township, and village institutions and breaking down the previously fragmented system. Specifically, resource devolution and technical assistance strengthen primary care capacity; medical insurance payment reforms (such as global budgets and surplus retention) create intrinsic incentives for first-contact care at the primary level; and information and resource-sharing centers improve coordination across the county health system.

However, these policy effects do not unfold linearly. The impact of CCMC construction on patient flow reflects two competing forces—patient diversion and patient return—and their dynamic balance determines the phased pattern of policy outcomes. In the early stage, county-level lead hospitals may, by virtue of their leading status and resource advantages, draw patients and qualified healthcare personnel away from grassroots facilities during the resource integration process; at the same time, the short-term resource reallocation and benefit adjustment of early reform may cause temporary fluctuations in grassroots operational efficiency, leading to an initial decline in the share of in-county visits. As CCMC construction deepens—with steady improvements in policy coverage, organizational tightness, and institutional safeguards-stable technical assistance, workforce devolution, and benefit-sharing mechanisms become established between county hospitals and primary facilities. Primary service capacity is substantially strengthened, the incentives of payment reform are gradually realized, and patient return to county facilities become the dominant trend. Accordingly:

*H1*: There is a U-shaped relationship between CCMC construction intensity and the relative share of in-county visits: the effect is negative in the early stages and turns positive after the inflection point.

Furthermore, the policy effects of CCMC construction may depend on initial conditions such as the external market environment and regional characteristics. On the one hand, the competitive structure of the external healthcare market moderates patient flow dynamics under public CCMC leadership; on the other, gradient differences in regional development produce uneven healthcare resource endowments and policy enforcement capacity across regions, suggesting that resource-constrained areas may have greater marginal scope for improvement. Accordingly:

*H2*: The impact of CCMC construction on the relative share of in-county visits varies significantly by medical market structure and regional development level.

## 3 Research design

### 3.1 Sample selection and data sources

#### 3.1.1 Sample selection

This study uses provinces as the unit of analysis, based on two main considerations. First, although CCMC construction is implemented at the county level, pilot designation, policy rollout, and coverage expansion are coordinated by provincial health authorities. Provinces differ systematically in policy coverage, organizational tightness, and progress of medical insurance payment reform, and these inter-provincial variations are the primary source of variation for our core explanatory variable (the construction intensity index). Second, systematic public statistics on county-level CCMC intensity are unavailable; constructing provincial-level composite indicators from policy documents and statistical materials is a feasible way to measure provincial implementation effort under current data constraints.

Following the policy provision in the *Guiding Opinions on Comprehensively Promoting the Construction of Tightly Integrated County-Level Medical Communities* (Guo Wei Ji Ceng Fa [2023] No. 41)—which states that “eligible municipal districts may follow these guidelines”-we define the treatment group for municipalities directly under the central government as follows. Beijing, Shanghai, and Tianjin establish district-level tightly integrated CCMCs only in suburban agricultural districts (where township health centers and village clinics exist), while organizing urban medical groups in central urban districts; Chongqing uniformly implements “county/district-level medical communities” across all its districts and counties, with reform design identical to that of ordinary counties. Accordingly, the CCMCs analyzed in this study include not only ordinary counties and county-level cities but also district-level tightly integrated CCMCs in agricultural municipal districts, all coded as the treatment group; purely central urban districts (without township health centers or village clinics) are excluded. This follows standard scholarly practice: an integrated network of “district-level lead hospital + township health centers + village clinics” is treated as the same policy shock, regardless of whether the administrative unit is named a county or an agricultural district.

#### 3.1.2 Data sources

All data are drawn from official public statistics and policy documents. Core indicators—outpatient visits, inpatient visits, and medical resources—come primarily from the *China Health Statistics Yearbook* (2015–2025). Macroeconomic variables such as population, economic development, and urbanization rates come from the *China Statistical Yearbook* (2015–2025). Government health expenditure and medical insurance fund data are collected from provincial statistical communiqués, provincial health development reports, and public disclosures by provincial healthcare security administrations. Lists of CCMC pilots, medical insurance payment reform pilots, and policy documents are drawn from provincial health commission websites and compiled manually.

### 3.2 Variable definitions

#### 3.2.1 Dependent variable

We use the relative share of in-county outpatient visits as the core dependent variable to measure patient flow directions and reflect the relative level of county-level service supply and patient retention across provinces. This indicator is defined as the ratio of total outpatient visits in county-level medical institutions within a province to total outpatient visits across all medical and health institutions in the province:

$$\begin{array}{l} \text{Relative share of in}-\text{county visits}=\\ \frac{\text{Total visits in county}-\text{level institutions within the province}}{\text{Total visits across}\;\mathrm{all}\;\text{medical and health institutions in the province}} \end{array}$$

Consistent with the “three-tier county linkage network” defined in Document Guo Ban Fa [2017] No. 32, county-level medical and health institutions fall into three categories: (1) county-level hospitals (general hospitals and TCM hospitals), which serve as regional hubs for complex cases and technical leadership; (2) primary care institutions (township health centers, community health centers/stations, and village clinics), which provide basic medical and public health services; and (3) county-level specialized public health institutions (county-level maternal and child health hospitals and specialized disease prevention institutions). All of these fall under the same county-level administrative unit and together form the integrated CCMC service network. Per the classification rules of the *China Health Statistics Yearbook*, out-of-county hospitals refer to institutions outside the county’s general and TCM hospitals (e.g., municipal, provincial, or cross-regional tertiary hospitals), which provide high-level care and absorb patients who leave the county.

The numerator counts visits across all county-level institutions in the province; the denominator counts all medical and health institutions in the province. Because of aggregate-level data limitations, cross-provincial patient flows (such as cross-provincial medical care) cannot be fully disaggregated at the individual level. At the provincial aggregate level, however, bidirectional or multilateral cross-provincial flows tend to offset one another. Moreover, the baseline model includes rigorous province and year fixed effects, which absorb time-invariant provincial structural differences. The relative share therefore provides a reasonable macro-level approximation of local patient retention and return trends at the county level.

We use the relative share of county-level visits for two reasons. First, it directly reflects the proportion of care delivered within the county, aligning with the tiered healthcare goal of raising county-level visit rates. Second, relative values neutralize cross-provincial differences in population size, market scale, and economic development, greatly improving comparability in the provincial panel. Owing to the hierarchical reporting system in national health statistics, grassroots visit counts may contain minor double-counting when aggregated county by county; provincial-level totals have already been deduplicated, so values slightly above 1 in a few provinces are within the expected statistical range.

Descriptive statistics show the indicator ranges from 0.323 to 0.845, with a stable distribution and no extreme outliers; the baseline regression therefore uses the raw calculated values. To ensure results do not depend on a single measure, robustness checks replace the dependent variable with per capita county-level visits. The relative shares of in-county inpatient visits, out-of-county outpatient visits, and out-of-county inpatient visits are calculated identically and are not repeated here.

#### 3.2.2 Explanatory variable

Traditional difference-in-differences models rely on 0–1 dummy variables that only identify whether a policy exists, making it difficult to capture year-by-year implementation depth and intensity. To address this, we construct a continuous CCMC Construction Intensity Index (CII) from three dimensions—policy coverage, organizational tightness, and institutional safeguards—using an objective entropy-weighting method. All indicators are positive: a higher CII indicates stronger overall CCMC construction and deeper reform in a province.

The rationale for each indicator is as follows:

1. Policy coverage is measured by the share of pilot counties. Per Document Guo Wei Ji Ceng Han [2019] No. 121, national CCMC pilots began in 2019 and expanded until near-universal coverage; this indicator captures the macro scale and speed of provincial rollout.
2. Organizational tightness is measured by the share of tightly integrated pilot counties. Per the evaluation criteria in Document Guo Wei Ban Ji Ceng Fa [2020] No. 12, a qualified tightly integrated CCMC must establish responsibility, management, service, and benefit integration under unified governance; this indicator distinguishes in-name-only pilots from substantively integrated reforms.
3. Institutional safeguards are measured by progress in medical insurance payment reform. Per Document Guo Wei Ban Ji Ceng Fa [2023] No. 41, which mandates global budgets, surplus retention, and reasonable over-expenditure sharing, we use the share of counties implementing bundled medical insurance payments to reflect supporting institutional development.

Raw data for all indicators come from official documents issued by provincial health commissions and healthcare security bureaus, ensuring objective and traceable sources. The indicator system is summarized in Table 2.

**Table 2:** Indicator system and measurement methods for compact county-level medical community construction intensity.

Dimension	Indicator	Measurement	Weight
Policy coverage	Share of pilot counties	Number of pilot counties / Total number of counties in the province	0.341
Organizational tightness	Share of tightly integrated pilot counties	Number of tightly integrated pilot counties / Number of CCMC pilot counties in the province	0.337
Institutional safeguards	Progress of medical insurance payment reform	Number of counties implementing bundled medical insurance payment / Total number of counties in the province	0.322

All indicators are positive. A higher value indicates greater CCMC construction intensity.

To avoid bias from subjective weighting, this study uses the entropy method to synthesize the multidimensional baseline indicators. The calculation steps are as follows:

Step 1: Indicator standardization. All baseline indicators in Table 2 are positive. To eliminate the influence of scale and extreme values, the raw data are standardized as follows^1^:

$${x_{\mathrm{ij}}}^{\prime }=\frac{x_{\mathrm{ij}}-\min(x_{j})}{\max(x_{j})-\min(x_{j})}$$

Step 2: Calculation of indicator weights. The characteristic proportion $p_{\mathrm{ij}}$ of province i under indicator j is calculated as:

$$p_{\mathrm{ij}}=\frac{{x_{\mathrm{ij}}}^{\prime }}{\sum_{i=1}^{n}{x_{\mathrm{ij}}}^{\prime }}$$

Step 3: Compute the normalized Shannon entropy and discrimination coefficient. The normalized Shannon entropy $e_{j}$ and information utility value (differentiation coefficient) $g_{j}$ of indicator j are calculated as:

$$e_{j}=-\frac{1}{\ln(n)}\sum_{i=1}^{n}p_{\mathrm{ij}}\ln(p_{\mathrm{ij}}),\;\;g_{j}=1-e_{j}$$

where n is the total number of sample provinces. The smaller the normalized Shannon entropy of an indicator, the greater its degree of differentiation and the more information it provides.

Step 4: Weight determination and composite index synthesis. The objective weight $w_{j}$ of each indicator is calculated, and weighted summation yields the CCMC construction intensity composite index (CII) for each province:

$$w_{j}=\frac{g_{j}}{\sum_{j=1}^{m}g_{j}},\;\;\mathrm{CII}=\sum_{j=1}^{m}w_{j}\cdot {x_{\mathrm{ij}}}^{\prime }$$

The weights reported in Table 2 are the objective weights derived from the entropy method. As the results show, the three dimensions are fairly balanced, with weights of 0.341 for policy coverage, 0.337 for organizational tightness, and 0.322 for institutional safeguards.

These weights objectively reflect data dispersion and information contribution: rather than being assigned subjectively, they are derived through normalized Shannon entropy based on the actual distribution of provincial data across the three dimensions. The magnitude of each weight reflects, to some extent, the information contribution of that dimension-a larger weight indicates greater inter-provincial variation and richer effective information, and thus plays a more prominent role in capturing overall CCMC implementation depth.

Specifically, policy coverage has the highest weight (0.341), suggesting that the breadth of pilot rollout is the primary factor driving differences in CCMC construction across provinces, with notable variation in administrative mobilization and pace of implementation. Organizational integration ranks second (0.337), highlighting that substantive integration quality is a key dimension of reform depth and helps reduce the influence of formalistic listing. Institutional safeguards ranks third (0.322), reflecting that bundled medical insurance payment—a deeper reform area—also constitutes a core institutional constraint and a source of differentiation across provinces.

#### 3.2.3 Control variables

To mitigate omitted-variable bias and more cleanly identify the net effect of CCMC construction intensity on patient flow within counties, we follow Chen Ming-sheng (2018) and Andersen’s behavioral model of health services use (predisposing characteristics, enabling resources, and need factors) in selecting provincial control variables across five dimensions: economic development, demographics, medical resources, fiscal capacity, and disease structure (42). The variables are described below.

Economic development and demographic structure. We include the log of GDP per capita, net migration rate, and population aging rate. Provincial economic development tends to shape residents’ overall ability to pay and preferences for care quality: residents in wealthier areas are more likely to travel to higher-level facilities, so GDP per capita is closely related to cross-region care-seeking. Population mobility alters the local patient base and supply–demand balance, which in turn affects the geographic distribution of visits. In addition, older adults account for a large share of chronic disease and inpatient demand and tend to have a noticeable effect on visit volumes across facility levels. Including these variables helps absorb the influence of broad structural differences in macroeconomic conditions, migration, and aging on patient care-seeking.

Medical resources and institutional capacity. We include the log of hospital beds per 1,000 people, per capita government health expenditure, and the log of cumulative medical insurance fund balances. Physical supply capacity-particularly bed availability—shapes the physical capacity and spatial distribution of facilities across levels. If not controlled, inter-provincial differences in bed supply could be misattributed to CCMC policy effects. Fiscal investment and insurance fund balances also provide important macro-level support and financial backing for primary care, and their adequacy influences how effectively payment reforms guide patient flow.

Disease structure. We include the share of discharges from internal medicine and oncology departments as a control for disease-mix heterogeneity. Provinces differ in their epidemiological profiles and case severity: conditions such as malignant tumors often require referral to provincial capitals or higher-level hospitals, generating upward cross-region flows. Disease mix therefore accounts for a substantial share of the observed differentiation in care-seeking levels. Controlling for this variable helps isolate the objective influence of case-severity differences on patient flow, providing a more reliable basis for identifying the net effect of CCMC policy.

Together, these controls are theoretically linked to patient flow directions and help reduce omitted-variable bias, strengthening the robustness of the core estimates.

#### 3.2.4 Descriptive statistics

The empirical analysis uses a provincial panel dataset covering 31 provinces, municipalities, and autonomous regions in China from 2014 to 2024, yielding 341 province-year observations. The sample covers approximately 2,800 county-level administrative units nationwide, with an average of about 90 counties per province. By the end of 2024, China had 1.0936 million medical institutions in total, of which 95.2% were grassroots facilities (including township health centers, community health centers/stations, and village clinics). The number of institutions varies across provinces in line with population size, with more facilities in the populous central and eastern provinces and fewer in the smaller western provinces.

To mitigate heteroskedasticity and scale differences, continuous variables such as GDP per capita and beds per 1,000 people are winsorized at the 1st and 99th percentiles and then log-transformed. Variance inflation factor (VIF) tests yield a mean of 3.72, well below the conventional threshold of 10, indicating that multicollinearity is not a serious concern. Descriptive statistics for the main variables are reported in Table 3.

**Table 3:** Descriptive statistics of main variables.

Variable	*N*	Mean	Std. Dev.	Min	Max
Relative share of in-county outpatient visits	341	0.679	0.127	0.323	0.845
Relative share of in-county inpatient visits	341	0.890	0.398	0.006	1.679
Relative share of out-of-county hospital outpatient visits	341	0.300	0.125	0.131	0.650
Relative share of out-of-county hospital inpatient visits	341	0.503	0.190	0.210	0.984
CCMC construction intensity index (CII)	341	0.395	0.374	0.000	1.000
GDP per capita (yuan)	341	69,055.38	34,727.67	25,101	228,167
Net migration rate	341	0.038	0.192	−0.167	0.695
Population aging rate	341	0.118	0.031	0.057	0.189
Per capita government health expenditure (yuan)	341	1,418.941	734.928	610.119	5,847.838
Hospital beds per 1,000 population	341	6.189	1.188	3.532	9.227
Cumulative medical insurance fund balance (10,000 yuan)	341	941,936.2	10,200,000	300,219	65,000,000
Share of internal medicine discharges	341	0.278	0.045	0.208	0.416
Share of oncology discharges	341	0.049	0.022	0.005	0.116

*N* = 341 province-year observations. Continuous variables are winsorized at the 1st and 99th percentiles.

### 3.3 Econometric model specification

To examine the impact of CCMC construction intensity on patient flow directions, we begin with a baseline two-way fixed-effects linear model:

$$Y_{\mathrm{it}}=\alpha +\beta_{1}\mathrm{CI}I_{\mathrm{it}}+\gamma X_{\mathrm{it}}+\mu_{i}+\lambda_{t}+\varepsilon_{\mathrm{it}}$$

where $Y_{\mathrm{it}}$ is the relative share of in-county outpatient visits in province i in year t ; $\mathrm{CI}I_{\mathrm{it}}$ is the CCMC construction intensity index; $X_{\mathrm{it}}$ is a vector of control variables; $\mu_{i}$ and $\lambda_{t}$ denote the province fixed effects and year fixed effects, respectively; and $\varepsilon_{\mathrm{it}}$ is the random error term.

Given that the effect of CCMC construction on patient flow may not be linear, we add a quadratic term to the model to allow for nonlinear relationships:

$$Y_{\mathrm{it}}=\alpha +\beta_{1}\mathrm{CI}I_{\mathrm{it}}+\beta_{2}\mathrm{CC}I_{\mathrm{it}}^{2}+\gamma X_{\mathrm{it}}+\mu_{i}+\lambda_{t}+\varepsilon_{\mathrm{it}}$$

where $\mathrm{CI}I_{\mathrm{it}}^{2}$ is the squared term of the construction intensity index, intended to capture a potential U-shaped or inverted-U pattern. A significantly negative $\beta_{1}$ together with a significantly positive $\beta_{2}$ would indicate a U-shaped relationship, whereas a significantly positive $\beta_{1}$ together with a significantly negative $\beta_{2}$ would indicate an inverted-U relationship.

To more credibly identify the policy effect and mitigate omitted-variable bias, the model includes both province fixed effects $\mu_{i}\;$ and year fixed effects $\;\lambda_{t}$ . Province fixed effects absorb time-invariant structural differences across provinces—such as geographic location, medical resource endowments, and regional healthcare culture. Year fixed effects absorb common national shocks, such as macroeconomic fluctuations and nationwide health system reforms, that affect all provinces in a given year. Given that error terms may be serially correlated and heteroskedastic within provinces over time, we cluster standard errors at the province level to ensure valid statistical inference.

## 4 Empirical results and analysis

### 4.1 Benchmark regression results

#### 4.1.1 Effects on in-county outpatient and inpatient care

Table 4 reports the baseline regression results. All specifications include province and year fixed effects to absorb time-invariant regional heterogeneity and common macro-level trends. The overall R^2^ is approximately 0.97, reflecting the strong persistence inherent in provincial macro-panel data. The within R^2^ values are 0.158 and 0.119 for the two columns, respectively. This suggests that, after netting out the long-run baseline differences absorbed by fixed effects, the model still captures a meaningful share of the variation in province-level responses to CCMC rollout, which helps ease concerns about purely mechanical associations or overfitting.

**Table 4:** Baseline regression results.

Variable	(1) In-county outpatient visit share	(2) In-county inpatient visit share
CII	−0.139^***^ (0.046)	0.227 (0.219)
CII^2^	0.135^***^ (0.037)	−0.124 (0.146)
GDP per capita (log)	−0.056 (0.071)	−0.254 (0.272)
Net migration rate	0.016 (0.105)	−0.131 (0.460)
Aging rate	−0.167 (0.191)	1.405 (0.842)
Per capita government health expenditure (log)	−0.030 (0.024)	−0.130 (0.159)
Beds per 1,000 population (log)	−0.124^**^ (0.054)	−0.082 (0.192)
Cumulative insurance fund balance (log)	0.023 (0.022)	0.139^*^ (0.070)
Internal medicine discharge share	0.074 (0.277)	1.008 (0.803)
Oncology discharge share	0.595 (0.578)	−1.107 (1.807)
Constant	1.364^*^ (0.780)	2.162 (3.125)
Province FE	Yes	Yes
Year FE	Yes	Yes
Observations	341	341
R^2^	0.978	0.968
Within R^2^	0.158	0.119

Cluster-robust standard errors at the province level are in parentheses. ^***^ *p* < 0.01, ^**^ *p* < 0.05, ^*^ *p* < 0.1. The same notation applies to all subsequent tables.

Looking at the outpatient visit results in column (1), the coefficient on the linear CII term is −0.139 (*p* < 0.01), and the coefficient on the squared term is 0.135 (*p* < 0.01), indicating a statistically significant U-shaped relationship. The estimated turning point is approximately 0.515, which falls within the observed range of the CII. In the early stage of construction, the share of in-county visits declines as CII rises, before increasing once the turning point is crossed. We further conduct a formal U-test to verify the statistical robustness of this U-shaped relationship.

Column (2) shows that neither the CII term nor its square has a statistically significant effect on the relative share of in-county inpatient visits. This may reflect the fact that inpatient care decisions are shaped by a more complex set of factors—including illness severity and insurance reimbursement rules—so CCMC effects on inpatient utilization may take longer to materialize. This pattern is consistent with recent evidence suggesting that CCMC effects on chronic-disease inpatient care and cost control operate with a lag (39).

Among the control variables, the coefficient on (log) beds per 1,000 population is −0.124 (*p* < 0.05), suggesting that greater bed supply does not, on its own, raise the in-county visit share. A plausible explanation is that newly added beds tend to be concentrated at county-level hospitals and above, which may reinforce the upward concentration of patients toward higher-level facilities.

Figure 1 displays the fitted relationship between CII and the relative share of in-county outpatient visits. When CII is below approximately 0.515, the in-county visit share declines as construction intensity increases. Once this turning point is crossed, the relationship shifts from negative to positive, and the in-county visit share gradually rebounds—providing visual confirmation of the U-shaped pattern.

**Figure 1:**
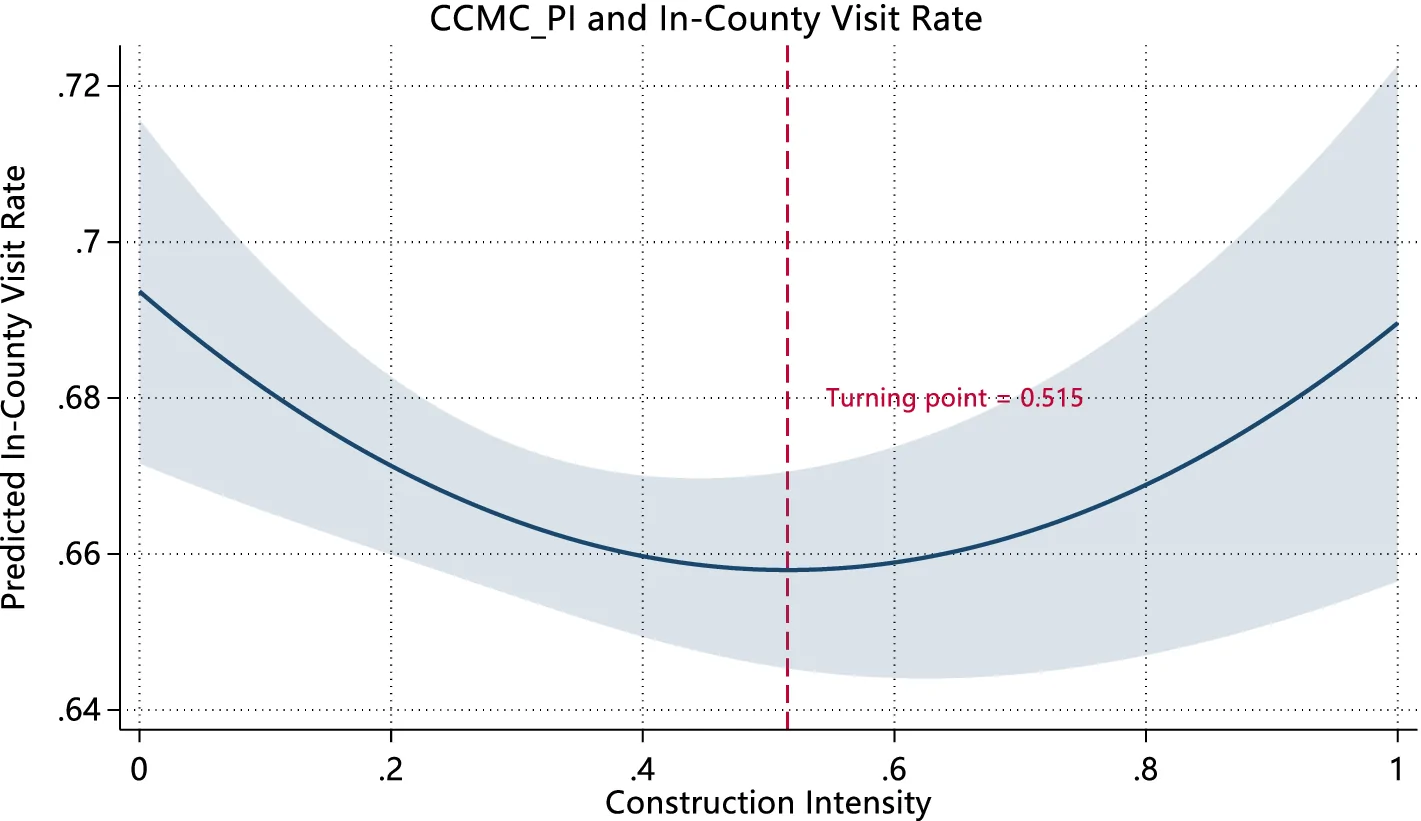
U-shaped relationship between medical community construction intensity and the relative scale of in-county medical visits.

To further verify the nonlinear relationship and rule out potential outlier-driven results, we systematically examined the distribution of observations on either side of the inflection point and the influence of extreme values. Of the 341 province-year observations in the full sample, 203 fall to the left of the inflection point (CII ≤ 0.515) and 138 fall to the right (CII > 0.515), suggesting that the “patient retention” phase on the right side has reasonably balanced sample support.

We also conducted right-tail sensitivity checks. Given that CII values are heavily clustered near zero—accounting for over 40% of the sample, reflecting provinces in the early or pre-pilot stage—we focus on one-sided trimming of the top 5% and top 10% of observations in the CII distribution. As reported in Table 5, after excluding these upper-tail observations, the regressions retain 303 and 294 observations respectively, with the number of clusters unchanged, satisfying large-sample asymptotic properties. The signs and statistical significance of both the linear and squared CII coefficients remain stable, suggesting that the U-shaped association is not spuriously driven by a small number of high-value observations.

**Table 5:** Robustness of the U-shaped relationship after excluding extreme values.

Variable	Baseline full sample (1)	Excluding top 5% percentile (2)	Excluding top 10% percentile (3)
CII	−0.139^***^ (0.046)	−0.108^**^ (0.048)	−0.108^**^ (0.046)
CII^2^	0.135^***^ (0.037)	0.153^***^ (0.042)	0.157^***^ (0.039)
Inflection point	0.515	0.350	0.345
Control variables	Yes	Yes	Yes
Province FE	Yes	Yes	Yes
Year FE	Yes	Yes	Yes
Observations	341	303	294
R^2^	0.978	0.982	0.983
WithR^2^	0.158	0.184	0.178

Columns (2) and (3) report estimates based on one-sided trimming of CII values above the 95th and 90th percentiles, respectively (i.e., right-tail trimming). Because CII values are heavily clustered in certain ranges, the number of observations removed at these percentile thresholds is slightly larger than 5 and 10% of the full sample, leaving 303 and 294 observations, respectively. Cluster-robust standard errors at the province level are reported in parentheses. *** *p* < 0.01, ** *p* < 0.05, * *p* < 0.1. The same notation applies to subsequent tables.

At the same time, comparing estimates across sample specifications reveals that the inflection point is somewhat sensitive to sample composition: as upper-tail observations are removed, the estimated turning point shifts from 0.515 in the full sample to 0.350 and 0.345, respectively. This indicates that the threshold for the transition from patient diversion to patient retention is not a fixed absolute value, but rather falls within an approximate range of [0.35, 0.52]—roughly corresponding to the 40th to 60th percentiles of the CII distribution. The existence of this range suggests that, while the exact intensity at which the association switches sign varies somewhat across subsamples, the phased pattern of “initial diversion followed by later retention” remains intact. In practice and in future policy evaluation, this turning point should be viewed as a dynamic transition zone rather than a rigid cutoff, providing more flexible guidance for context-specific CCMC implementation.

To further verify the statistical robustness of the U-shaped relationship, we conduct the U-test proposed by Lind and Mehlum (44), with results reported in Table 6. The estimated turning point is 0.515, which falls within the observed range of CII. The slope on the left side of the turning point is −0.139 (*t* = −3.04, *p* = 0.002), and the slope on the right side is 0.131 (*t* = 2.80, *p* = 0.004). The two slopes have opposite signs and are both significant at the 1% level. The overall U-test yields a t-statistic of 2.80 (*p* = 0.004), rejecting the null hypothesis of a monotonic or inverted-U relationship and supporting the presence of a U-shaped pattern.

**Table 6:** U-shaped relationship test.

Interval	Range	Slope	*t*-statistic	*p*-value
Left side (x < 0.515)	(0, 0.515)	−0.139^***^	−3.04	0.002
Right side (x > 0.515)	(0.515, 1)	0.131^***^	2.80	0.004
Turning point	0.515	—	—	—
Overall U-test	—	—	2.80	0.004

^**^ *p* < 0.05, ^***^ *p* < 0.01. The null hypothesis is a monotonic or inverted-U relationship; the alternative is a U-shaped relationship. The turning point falls within the observed range of the CII, and the slopes on both sides have opposite signs and are statistically significant, supporting the U-shaped pattern.

These results suggest that the effect of CCMC construction on patient flow follows a nonlinear path—first declining and then improving. In the early stage, during the transitional period of institutional alignment and resource restructuring, some patients may initially seek care at higher-level hospitals outside the county, causing a temporary decline in the in-county visit share. As substantive integration deepens and primary care capacity strengthens, the benefits of reform gradually materialize, and patients begin to return to county-level facilities.

#### 4.1.2 The “diversion–return” mechanism between county hospitals and primary care

Having established the U-shaped relationship for overall in-county visits, we next disaggregate in-county care into two tiers-primary care institutions and county hospitals—to examine the differential effects of CCMC construction on the composition of county-level care. The results are reported in Table 7.

**Table 7:** Disaggregated results for in-county care utilization.

Variable	Proportion of Primary-Level Medical Visits	Proportion of Medical Visits to County and County-Level City Hospitals		
		County and county-level city hospitals	Maternal and child health hospitals	Specialized disease prevention and treatment hospitals
CII	−0.222^***^ (0.074)	0.213^***^ (0.070)	0.011^*^ (0.006)	−0.002^*^ (0.001)
CII^2^	0.216^***^ (0.053)	−0.208^***^ (0.051)	−0.009^*^ (0.005)	0.001 (0.001)
Controls	Yes	Yes	Yes	Yes
Province FE	Yes	Yes	Yes	Yes
Year FE	Yes	Yes	Yes	Yes
Observations	341	341	341	341
R^2^	0.931	0.932	0.949	0.947
Within R^2^	0.129	0.127	0.246	0.196

Cluster-robust standard errors at the province level are in parentheses. ^***^ *p* < 0.01, ^**^ *p* < 0.05, ^*^ *p* < 0.1. The same notation applies to all subsequent tables.

Column (1) shows that CII has a significant U-shaped effect on the primary care visit share: the linear term is −0.222 (*p* < 0.01) and the squared term is 0.216 (*p* < 0.01). In other words, the primary care visit share first declines as CII increases, then rises after the turning point. The U-test further confirms the statistical robustness of this U-shaped pattern (*t* = 2.99, *p* < 0.01). In contrast, column (2) shows that CII has a significant inverted-U effect on the county hospital visit share: the linear term is 0.213 (*p* < 0.01) and the squared term is −0.208 (*p* < 0.01). The county hospital share first rises during early construction, then gradually declines after the turning point. The U-test also supports this inverted-U relationship (*t* = 3.04, *p* < 0.01).

Together, these results point to a dynamic reallocation process between county hospitals and primary care facilities: an initial upward concentration of patients followed by a subsequent downward diversion. In the early stage, patients move out of primary care, so the county hospital share rises while the primary care share falls. As integration deepens, county hospitals begin to refer recovering and chronic-disease patients back to grassroots facilities, so the county hospital share declines and the primary care share rebounds, gradually optimizing the composition of care within the county. In addition, the visit shares of maternal and child health hospitals and specialized disease prevention institutions show relatively little change, suggesting that CCMC effects operate primarily through the interaction between county hospitals and primary care facilities.

#### 4.1.3 Robustness check: out-of-county care

To further rule out the possibility that the initial decline in in-county visit share simply reflects a sustained outflow of patients to hospitals outside the county, we next examine how CCMC construction affects the relative share of visits and inpatient stays at out-of-county hospitals. The results are reported in Table 8.

**Table 8:** Out-of-county care utilization.

Variable	(1) Out-of-county outpatient visit share	(2) Out-of-county inpatient visit share
CII	0.131^***^ (0.043)	−0.098^*^ (0.053)
CII^2^	−0.129^***^ (0.035)	0.049 (0.039)
Controls	Yes	Yes
Province FE	Yes	Yes
Year FE	Yes	Yes
Observations	341	341
R^2^	0.979	0.978
Within R^2^	0.157	0.174

Cluster-robust standard errors at the province level are in parentheses. ^***^ *p* < 0.01, ^**^ *p* < 0.05, ^*^ *p* < 0.1. The same notation applies to all subsequent tables.

Column (1) shows that CII has a significant inverted-U effect on the out-of-county outpatient visit share: the share rises during early construction, then gradually declines after the turning point at approximately 0.51. Figure 2 illustrates this inverted-U pattern. This result mirrors the U-shaped relationship for overall in-county visits (Figure 1) and the disaggregated within-county results (Figures 3, 4): in the early stage, out-of-county patient flows increase; as CCMC construction deepens, patients begin to return to the county, the county hospital share declines, and the primary care share rebounds, gradually optimizing the composition of care-seeking within the county.

**Figure 2:**
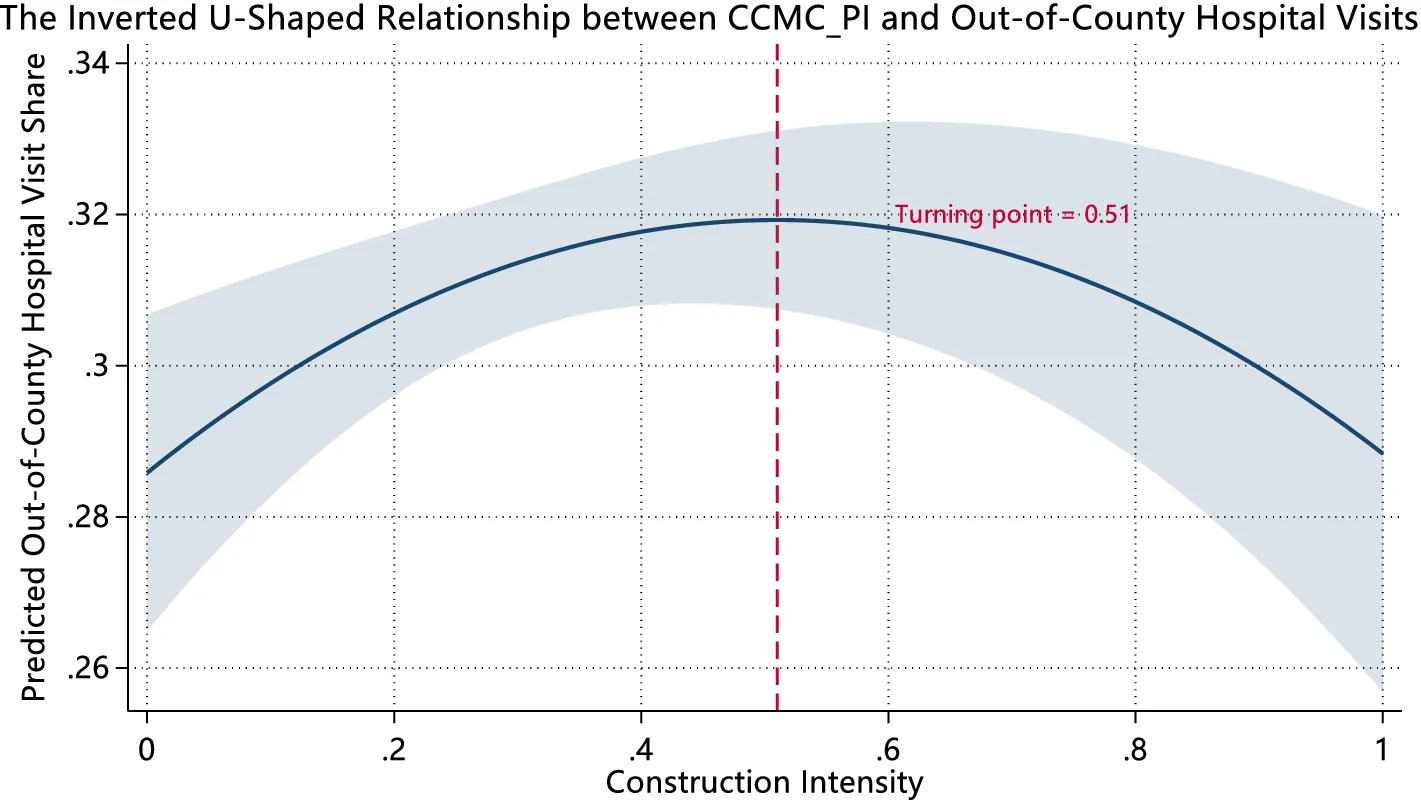
Inverted U-shaped relationship between medical community construction intensity and the proportion of out-of-county hospital medical visits.

**Figure 3:**
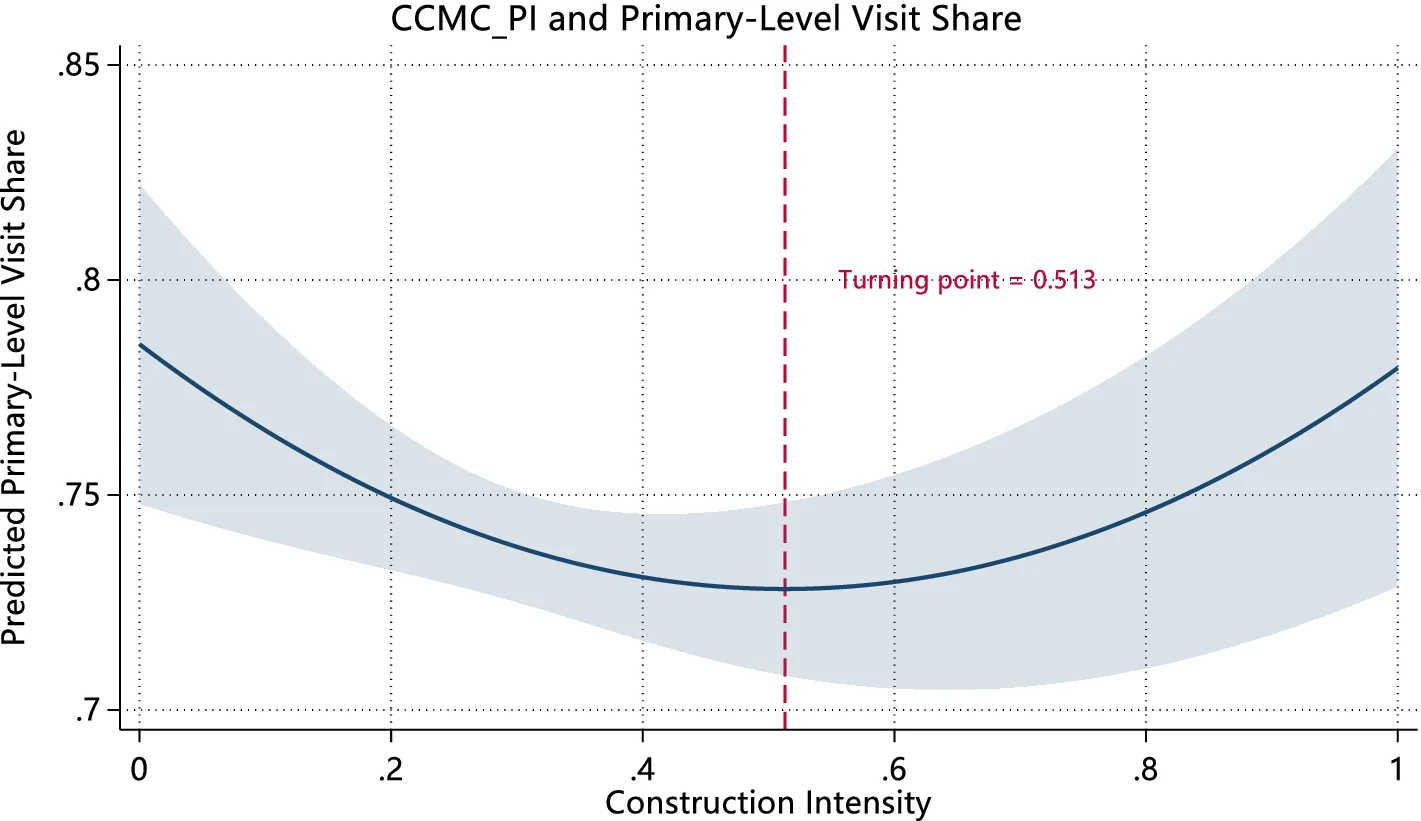
U-shaped relationship between medical community construction intensity and the proportion of primary-level medical visits.

**Figure 4:**
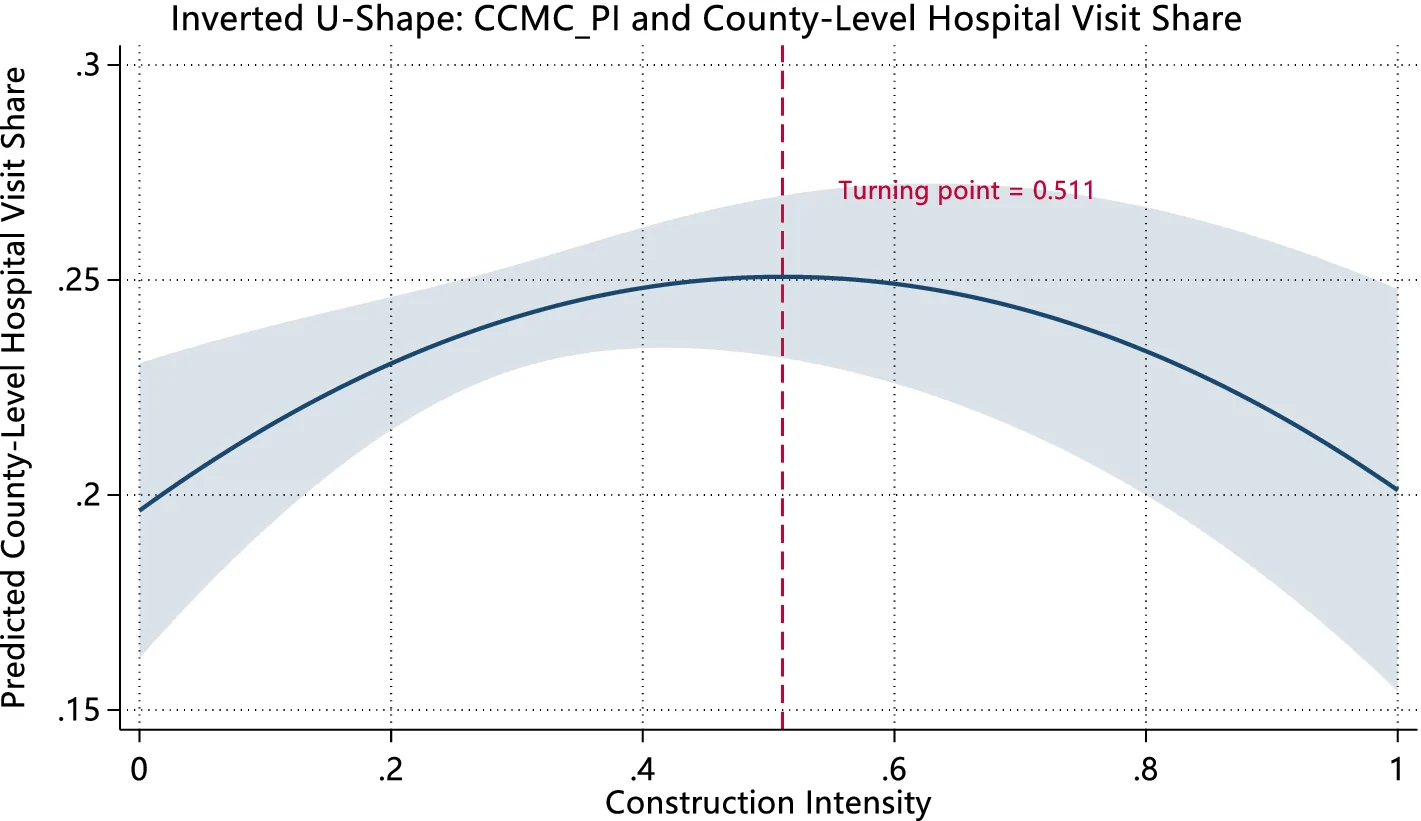
Inverted U-shaped relationship between medical community construction intensity and the proportion of medical visits to county and county-level city hospitals.

For inpatient care, column (2) shows that the linear CII term has a coefficient of −0.098 (*p* < 0.01), while the squared term is not statistically significant. This suggests that CCMC construction is associated with a roughly linear decline in out-of-county inpatient visits.

Taken together, the results in Tables 4, 7, and 78indicate that the initial decline in the in-county visit share is not driven by a permanent exodus of patients to out-of-county hospitals. Rather, it reflects the combined effect of an early “diversion effect”—whereby county hospitals draw patients away from grassroots facilities—and a temporary increase in out-of-county flows. As construction deepens, patients gradually return to the county, and the overall pattern of care-seeking becomes more balanced.

### 4.2 Province-specific linear trends

To further address potential confounding from unobserved province-specific factors that evolve linearly over time—such as regional development trajectories or differences in the pace of health policy rollout—we add province-specific linear time trends to the two-way fixed-effects model, as reported in Table 9. After including these trends, the coefficients on CII and CII^2^ are −0.031 and 0.027, respectively. While the signs remain consistent with the baseline estimates, neither coefficient reaches statistical significance. Statistically, this likely reflects the fact that province-specific linear trends are highly correlated with slowly evolving variables such as CCMC construction intensity, and absorb a substantial share of within-province variation—within R^2^ declines from 0.157 to 0.048—leaving a narrower range of identifiable variation and reducing estimation precision.

**Table 9:** Robustness check: province-specific linear trends.

Variable	(1) Baseline	(2) With province-specific trends
CII	−0.139^***^ (0.046)	−0.031 (0.038)
CII^2^	0.135^***^ (0.037)	0.027 (0.027)
Controls	Yes	Yes
Province FE	Yes	Yes
Year FE	Yes	Yes
Province × year linear trends	No	Yes
Observations	341	341
R^2^	0.979	0.994
Within R^2^	0.157	0.048

Cluster-robust standard errors at the province level are in parentheses. ^***^ *p* < 0.01, ^**^ *p* < 0.05, ^*^ *p* < 0.1. The same notation applies to all subsequent tables.

However, we note objectively that this result also implies the U-shaped nonlinear association identified in this study may be partly driven by long-term macro time trends, rather than being determined solely by the nonlinear dynamics of the policy itself. Overall, although we observe a U-shaped association in the baseline specification, this association does not remain statistically significant under more stringent trend controls. We therefore retain the two-way fixed-effects model as our baseline specification and present the province-specific trend test as a sensitivity check under a more demanding scenario. All academic conclusions and causal inferences are drawn within this acknowledged robustness boundary.

### 4.3 Robustness checks

To verify the robustness of the baseline findings and address concerns about variable measurement, sample selection, and model specification, we conduct a series of robustness checks along five dimensions. The results are reported in Table 10. First, we re-estimate the CII using two alternative weighting approaches—principal component analysis (PCA) and the CRITIC method. The signs, significance levels, and turning points of the key coefficients remain broadly consistent with the baseline estimates, suggesting that the findings are not sensitive to the choice of weighting method. Second, we replace the dependent variable with per capita in-county visits. The U-shaped effect of CCMC construction remains statistically significant. Third, we exclude the four directly administered municipalities (Beijing, Tianjin, Shanghai, and Chongqing) from the sample to address concerns that these special cases might drive the results. The core findings remain unchanged. Fourth, we further control for the visit shares of tertiary hospitals and private hospitals to account for differences in medical market structure. The baseline effect remains robust, ruling out alternative explanations based on market characteristics. Fifth, we conduct a permutation-based placebo test with 500 random assignments. Figures 5, 6 show the distribution of estimated coefficients from these random assignments. The actual estimates lie well outside the distribution of placebo coefficients, which helps alleviate concerns about omitted variable bias and random confounding.

**Table 10:** Robustness check results.

Variable	(1) PCA index	(2) CRITIC index	(3) Per capita visits	(4) Excluding municipalities	(5) Controlling for market structure
CII	−0.138^***^ (0.046)	−0.150^***^ (0.051)	−2.295^**^ (1.042)	−0.114^**^ (0.043)	−0.114^***^ (0.037)
CII^2^	0.135^***^ (0.038)	0.141^***^ (0.038)	2.124^***^ (0.771)	0.117^***^ (0.042)	0.113^***^ (0.039)
Controls	Yes	Yes	Yes	Yes	Yes
Province FE	Yes	Yes	Yes	Yes	Yes
Year FE	Yes	Yes	Yes	Yes	Yes
Observations	341	341	341	297	341
R^2^	0.978	0.978	0.913	0.948	0.952
Within R^2^	0.157	0.158	0.157	0.095	0.156

Province-clustered robust standard errors are in parentheses. ^***^ *p* < 0.01, ^**^ *p* < 0.05, ^*^ *p* < 0.1. The same notation applies to all subsequent tables.

**Figure 5:**
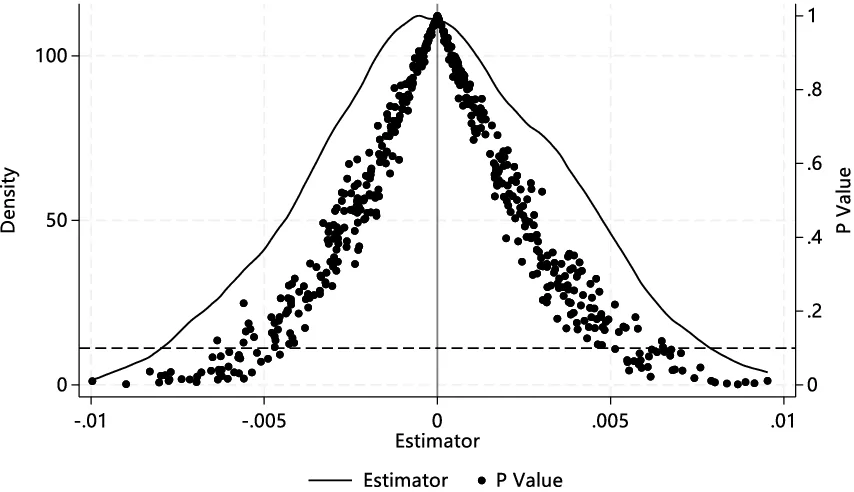
Placebo test results (linear term coefficient).

**Figure 6:**
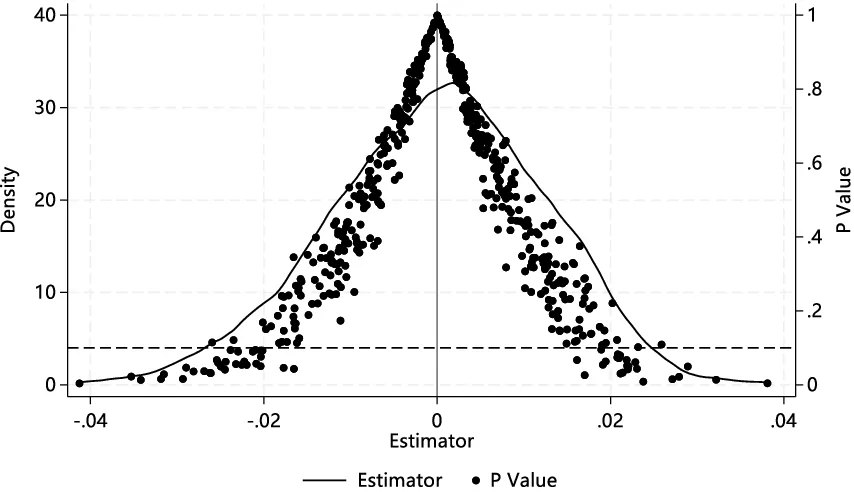
Placebo test results (squared term coefficient). The solid black line denotes the kernel density of the placebo estimates, while the hollow circles represent the *p*-values from each permutation. The gray solid line marks zero, and the black dashed line marks *p* = 0.1. The actual estimates (linear term = −0.139, squared term = 0.135) fall outside the central range of the placebo distribution, suggesting that the baseline results are unlikely to be driven by random chance.

Taken together, these checks suggest that the U-shaped relationship between CCMC construction intensity and the in-county visit share is robust to alternative measures, sample restrictions, and model specifications.

### 4.4 Sensitivity analysis

To examine whether the U-shaped nonlinear relationship depends on the specific index construction and weighting scheme, we conduct sensitivity tests along two dimensions: correlation with individual sub-indicators and dimension-by-dimension regressions.

First, we examine the association between the composite CII and the three individual sub-indicators. As shown in Table 11, the Pearson correlation coefficients between CII and the share of pilot counties, the share of tightly integrated pilot counties, and the pace of medical insurance payment reform are all above 0.91, and the Spearman rank correlation coefficients are all above 0.85, with all coefficients significant at the 1% level. This suggests that the composite index is closely aligned with its underlying dimensions, supporting the construct validity of the CII measure.

**Table 11:** Correlation matrix between CII and sub-dimension indicators.

Variable	CII	pilot_rat	integ	drg_dip_ratio
CII	1			
pilot_rat	0.9430 (0.9782)	1		
integ	0.929 (0.8596)	0.8006 (0.8148)	1	
drg_dip_ratio	0.9104 (0.9454)	0.8284 (0.8878)	0.7483 (0.8642)	1

Main entries are Pearson correlation coefficients; Spearman rank correlations are in parentheses. All coefficients are based on 341 observations and are significant at the 1% level.

To examine whether the U-shaped nonlinear relationship depends on the specific index construction and weighting scheme, we disaggregate the CII into its three sub-dimensions—policy coverage, organizational integration, and institutional safeguards—and re-estimate the nonlinear model using each dimension separately, as reported in Table 12. The results for policy coverage (Model 2) and institutional safeguards (Model 4) both exhibit a significant U-shaped pattern, consistent with the baseline model (1) and supporting the robustness of the main findings.

**Table 12:** Sensitivity analysis: Sub-dimension regressions.

Variable	(1) Baseline CII	(2) Policy coverage	(3) Organizational tightness	(4) Institutional safeguards
CII	−0.139^***^ (0.046)	−0.095^**^ (0.038)	0.454 (1.634)	−0.266^**^ (0.111)
CII^2^	0.135^***^ (0.037)	0.083^***^ (0.031)	−0.215 (0.908)	0.188^**^ (0.080)
Controls	Yes	Yes	Yes	Yes
Province FE	Yes	Yes	Yes	Yes
Year FE	Yes	Yes	Yes	Yes
Observations	341	341	341	297
R^2^	0.978	0.978	0.978	0.978
Within R^2^	0.158	0.156	0.140	0.135

Province-clustered robust standard errors are in parentheses. ^***^ *p* < 0.01, ^**^ *p* < 0.05, ^*^ *p* < 0.1. The same notation applies to all subsequent tables.

Notably, neither the linear nor the squared coefficient on organizational integration (Model 3) reaches statistical significance, and the signs do not display the same U-shaped pattern as the baseline. This result carries practical and theoretical implications. On one hand, substantive organizational integration—personnel, financial, and operational—is the “deep end” of CCMC reform, and at the provincial annual panel level, this dimension has limited cross-sectional variation, with reform effects likely to emerge only over a longer time horizon. On the other hand, this finding may reflect a short-term mismatch between formal and substantive integration. In the early stages of CCMC construction, when nominal organizational affiliation and structural integration are achieved primarily through administrative fiat without supporting resource devolution and payment reform, restructuring incurs adjustment costs from internal resource reallocation and management authority shifts. Organizational tightening alone may not be sufficient to drive patient retention at the grassroots level. This suggests that progress on organizational integration, when pursued in isolation, may not independently generate patient backflow in the short term. It is only when structural reorganization is combined with expanded policy coverage and medical insurance payment reform that the combined institutional forces begin to drive patient retention within counties.

### 4.5 Heterogeneity analysis

#### 4.5.1 Market structure heterogeneity

The degree of competition in the healthcare market may shape how CCMC policies affect patient flow. To explore this, we split the sample into two groups based on whether a province’s private hospital share in the base year (2014) is above or below the sample median and estimate the model with interaction terms. The results are reported in Table 13.

**Table 13:** Heterogeneity by market structure (private hospital share).

Variable	Interaction model
CII	−0.064 (0.045)
CII^2^	0.039 (0.042)
CII × High Private	0.071^***^ (0.023)
CII^2^ × High Private	−0.019 (0.022)
Controls	Yes
Province FE	Yes
Year FE	Yes
Observations	341
R^2^	0.983
Joint F-test	*F* (2, 30) = 8.87^***^ (*p* = 0.0009)

Province-clustered robust standard errors are in parentheses. High Private is a dummy equal to 1 if the province’s private hospital share in 2014 is above the sample median. ^***^ *p* < 0.01, ^**^ *p* < 0.05, ^*^ *p* < 0.1. The same notation applies to all subsequent tables.

The joint F-test on the interaction terms yields a statistic of 8.87 (*p* = 0.0009), significant at the 1% level, suggesting that market structure moderates the nonlinear effect of CCMC construction. Specifically, the interaction on the linear CII term is positive and significant (0.071, *p* < 0.01), indicating that the baseline effect differs in provinces with a higher private hospital share.

A plausible interpretation is twofold. On the one hand, in regions with a more active private sector, the greater diversity of supply gives patients more alternatives, which may dampen the initial flow disruption during the early phase of CCMC rollout. On the other hand, market competition may also push public hospitals to improve efficiency, adding an external layer of adjustment to how CCMC policy translates into patient flow changes.

#### 4.5.2 Regional heterogeneity

Given the substantial differences in economic development, healthcare resource endowment, and policy implementation across China’s four macro-regions—eastern, central, western, and northeastern—we follow common practice in regional economics and environmental studies (40) and run separate regressions by region. The results are reported in Table 14. The estimates show a clear spatial gradient in the nonlinear effect of CCMC construction.

**Table 14:** Regional heterogeneity analysis.

Variable	(1) East	(2) Central	(3) West	(4) Northeast
CII	0.103 (0.065)	0.045 (0.046)	−0.450^*^ (0.231)	3.962 (3.373)
CII^2^	−0.023 (0.049)	0.023 (0.014)	0.333^*^ (0.159)	−3.436 (2.746)
Controls	Yes	Yes	Yes	Yes
Province FE	Yes	Yes	Yes	Yes
Year FE	Yes	Yes	Yes	Yes
Observations	110	66	132	33
R^2^	0.992	0.974	0.948	0.967
Within R^2^	0.350	0.708	0.288	0.609

Province-clustered robust standard errors are in parentheses. Given the small sample size for the Northeast (only 3 provinces), those estimates should be interpreted as exploratory and are kept for regional comparison only. ^***^ *p* < 0.01, ^**^ *p* < 0.05, ^*^ *p* < 0.1.

First, the western region exhibits a significant U-shaped relationship. This suggests that the marginal benefits of CCMC resource devolution are particularly pronounced in areas with weaker resource endowments and lower initial access to care. A plausible reason is that, given its vast territory and sparse population, western China has long faced a severe shortage of high-quality medical resources, and grassroots institutions face both an upward drain of talent and weak service capacity. While the early stage brings transitional frictions and some patient diversion, continued policy deepening—supported by central fiscal transfers and east–west collaboration—creates substantial room for improvement, allowing the shift from initial outflow to subsequent patient return to emerge relatively early.

Second, the core variables are not statistically significant in the eastern and central regions. These regions have stronger economic foundations, a greater concentration of large hospitals, more abundant high-quality resources, and better-developed primary care systems before the reform began. In areas with convenient transportation and a wide range of care options, the marginal effect of institutional integration through CCMCs may be more limited, and established patterns of cross-regional care-seeking are unlikely to shift dramatically in the short run, making the U-shaped turning point harder to detect statistically.

Third, the northeastern region has a relatively small sample (3 provinces × 11 years = 33 observations), which limits statistical power and widens standard errors. Its estimates should therefore be interpreted as exploratory and kept for regional comparison rather than as precise causal evidence.

Taken together, the U-shaped pattern is most pronounced in the western region, where the scope for marginal improvement is greatest, while the effect is weaker in the more resource-abundant eastern and central regions.

We note that, although our heterogeneity analyses are theoretically grounded in the Andersen behavioral model and institutional logic and examine multiple dimensions such as market structure and regional location, we have not applied traditional conservative corrections for multiple hypothesis testing. This may increase the risk of false positives associated with multiple comparisons. Accordingly, the multidimensional heterogeneity findings reported here should be regarded as primarily exploratory in nature and interpreted with due caution when drawing policy or academic implications. Future research, ideally with richer individual-level panel data or quasi-experimental designs, could conduct more stringent boundary checks on specific dimensions.

## 5 Further discussion: differential effects across primary care facilities

The preceding analysis shows that CCMC construction has a significant U-shaped nonlinear effect on the overall in-county visit share. However, primary care facilities are not homogeneous—township health centers, community health centers (stations), and village clinics differ substantially in their service roles, resource endowments, and patient volumes. CCMC construction may therefore affect these facility types differently. To shed more light on where the policy effects operate, we examine outcomes by facility type.

### 5.1 Township health centers

Table 15 shows that CII has a positive and marginally significant effect on per capita visits at township health centers (0.119, *p* < 0.1), on outpatient and emergency visits (0.111, *p* < 0.1), and on daily visits per physician (0.298, *p* < 0.1). This suggests that as CCMC construction deepens, township health centers improve on both service volume and operational efficiency.

**Table 15:** Effects on township health centers.

Variable	Township health centers		
	Ln (*Per Capita* Visits)	Ln (Outpatient & Emergency Visits)	Ln (Daily Visits Per Physician)
CII	0.119^*^ (0.069)	0.111^*^ (0.059)	0.298^*^ (0.159)
Control variables	Yes	Yes	Yes
Province FE	Yes	Yes	Yes
Year FE	Yes	Yes	Yes
Observations	341	341	335
R^2^	0.955	0.945	0.971
WithinR^2^	0.122	0.129	0.121

Province-clustered robust standard errors are in parentheses. ^***^ *p* < 0.01, ^**^ *p* < 0.05, ^*^ *p* < 0.1. The same notation applies to all subsequent tables.

As the linchpin between county hospitals and village clinics, township health centers are the primary tier in the vertical integration structure—they are the first recipients of resource devolution and patient diversion from county hospitals. Talent support and technical guidance from county hospitals first reach township health centers, and after the turning point, patients referred downward are also routed here, driving concurrent gains in both volume and efficiency. For example, evidence from Linxiang City, Hunan Province, indicates that township health centers saw a 36.8% year-on-year increase in service volume and a 3.84% increase in medical revenue after CCMC implementation.

### 5.2 Community health centers (and stations) and village clinics

Table 16 shows that, for community health centers, the coefficients on all outcome variables are positive but not statistically significant. In contrast, for village clinics, the coefficient on per capita visits is 0.209 and is significant at the 5% level (*p* < 0.05). This suggests that, compared with urban community health facilities, village clinics-the rural “safety net” of the healthcare system—appear to be the first to benefit from resource devolution as CCMC construction deepens. For example, in Wangmo County, Guizhou Province, village clinic visits increased by 24.66% year-on-year in 2024.

**Table 16:** Estimation results of the effects on community health centers (stations) and village clinics.

Variable	Community Health Centers		Community Health Stations	Village Clinics	
	Ln (*Per Capita* Visits)	Ln (Daily Visits Per Physician)	Ln (*Per Capita* Visits)	Ln (*Per Capita* Visits)	Ln (Outpatient & Emergency Visits)
CII	0.055 (0.066)	0.059 (0.177)	0.018 (0.038)	0.209^**^ (0.098)	0.033 (0.098)
Control variables	Yes	Yes	Yes	Yes	Yes
Province FE	Yes	Yes	Yes	Yes	Yes
Year FE	Yes	Yes	Yes	Yes	Yes
Observations	341	337	341	341	341
R^2^	0.988	0.904	0.894	0.938	0.956
WithinR^2^	0.268	0.164	0.12	0.23	0.176

Province-clustered robust standard errors are in parentheses. ^***^ *p* < 0.01, ^**^ *p* < 0.05, ^*^ *p* < 0.1. Data on daily visits per physician at community health stations are missing for some provinces.

The weaker results for community health centers may reflect a mismatch in service orientation: these facilities are mostly located in urban areas, whereas CCMC policy is primarily rural-oriented. At the same time, the total outpatient volume at village clinics has not yet shown a significant increase, possibly due to a small base or substitution effects at the aggregate level.

Taken together, the grassroots-level effects of CCMC construction exhibit clear structural heterogeneity. Township health centers show relatively widespread improvements in both service volume and operational efficiency; village clinics show localized gains in per capita visit volume; while urban community health centers, given their administrative and service positioning, respond relatively slowly. These findings suggest that the benefits of resource devolution currently concentrate mainly on the rural primary-care safety net, and that the capacity to absorb policy effects varies across tiers of grassroots institutions. This is consistent with the view that coordinated development of resources, medical services, and medical insurance plays a supporting role in grassroots health system restructuring (41). Future reforms should build on the progress of county-township integration and adopt differentiated strategies for village-level and other types of institutions, so as to remove institutional bottlenecks that impede the deep delivery of health services.

Although these multidimensional analyses reveal structural patterns in how different grassroots institutions benefit from CCMC construction, we note that examining multiple sub-categories—township health centers, village clinics, and community health centers—involves parallel testing across several outcomes. In the absence of formal multiple-comparison corrections, these results should be regarded as exploratory in nature. When interpreting the differential policy benefits across grassroots institution types, readers should take into account the potential statistical biases introduced by multiple testing. Further research, ideally using longer individual-level panel data or field-based investigations, is needed to validate and deepen these findings.

## 6 Conclusions and policy recommendations

This study examines tightly integrated county-level medical communities (CCMCs) within the broader global literature on integrated care, referral, and patient retention. International experience indicates that countries such as the United States and the United Kingdom primarily rely on general practitioner gatekeeping, diagnosis-related group (DRG) payment incentives, and integrated information systems to achieve orderly patient flow and long-term retention. However, existing international evidence draws largely on mature universal health systems, with relatively limited attention to the phased characteristics of progressive reforms in resource-constrained settings.

Using provincial panel data from China over 2014–2024, this paper constructs a multidimensional CCMC Construction Intensity Index (CII) covering three dimensions—policy coverage, organizational integration, and institutional safeguards—and employs a two-way fixed-effects model to examine the nonlinear association between tightly integrated CCMC construction and patient flow directions. The findings suggest that, at the macro level, CCMC development in China does not follow a simple linear improvement path, but rather exhibits a threshold-dependent pattern. This offers a new perspective on ongoing debates regarding the effects of integrated care systems and contributes empirical evidence on the phased evolution of reform in developing countries to the global integrated care literature. The main findings are as follows:

First, there is a U-shaped association between CII and the relative scale of in-county visits, though this association is attenuated under more demanding trend controls and the estimated inflection point shows some sensitivity to sample composition. At lower levels of construction intensity, patient diversion toward county hospitals accompanies the resource integration process; once intensity crosses the threshold, substantive integration and long-term mechanisms are gradually unleashed, and patient retention within counties begins to dominate. This pattern is mirrored by an inverted U-shaped trend in out-of-county care-seeking.

Second, the composition of medical visits within counties has evolved structurally: the share of grassroots visits follows a U-shaped pattern with CCMC construction, while the share of county hospital visits follows an inverted U-shaped pattern, reflecting a dynamic process of initial upward concentration followed by subsequent downward diversion.

Third, the observed patterns vary across regions and medical market structures. Heterogeneity analysis indicates that the U-shaped pattern is more pronounced in western regions and in areas with a higher density of private hospitals. We note that, given the lack of correction for multiple comparisons, these heterogeneity findings should be regarded as exploratory in nature and interpreted with due caution when drawing policy implications.

Fourth, the benefits within the grassroots sector are distributed unevenly. The effects are concentrated mainly in township health centers; village clinics show improvements in per capita visit volume, while urban community health institutions respond relatively slowly.

These findings suggest that the U-shaped nonlinear association identified in this study is attenuated under more stringent trend controls, and that the inflection point shows some sensitivity to sample composition. Moreover, the analysis based on provincial macro-panel data primarily reflects ecological-level macro correlations. Accordingly, the following policy recommendations are derived from the association results of the baseline model and warrant further validation using micro-level or richer data in future research. In practice, relevant policy design and adjustment may consider the following directions:

First, recognize the phased nature of reform and maintain continuity and long-term policy commitment. Local governments may consider recognizing that CCMC construction follows a threshold-dependent evolutionary path, and avoid dismissing reform achievements based on early-stage patient diversion toward county hospitals. A longer-term perspective may be adopted to continuously broaden policy coverage and deepen substantive integration, facilitating the transition from formal affiliation to genuine integration of interests and services, and helping provinces navigate the critical transition zone.

Second, adapt policies to local contexts and explore resource allocation strategies tailored to resource-constrained regions. Given the observed regional and market-structure heterogeneity, policy design and resource investment may be adjusted to local conditions. For central, western, and other areas with relatively scarce medical resources, modest fiscal transfer payments and technical support may be directed toward strengthening grassroots hardware capacity and workforce attractiveness, so as to gradually release the marginal improvement potential of CCMC construction in western regions. Drawing on existing research and international reform experiences, policymakers may also explore standardized general practitioner training and multi-site practice policies to stabilize the grassroots workforce and help alleviate shortages of qualified personnel and weak trust in primary care (1).

Third, position township health centers as important recipients of resource devolution and address bottlenecks in two-way referrals. In practice, township health centers may be designated as key hubs for receiving devolved resources and diverting patients within the vertical integration framework, supporting the policy goal of keeping common and frequent conditions at the primary level. To address institutional barriers where large hospitals may be reluctant to engage in two-way referrals due to economic incentives, measures such as dispatching attending physicians on a regular basis, streamlining green channels for upward and downward transfers, and refining global budget and surplus-retention payment incentives may be explored to mitigate interest-related resistance and enhance the service appeal and operational efficiency of township health centers (2).

Fourth, address grassroots capacity gaps at both the village and community levels and explore integrated medical-preventive care. Given the potentially differential benefits across grassroots institution types, policy implementation may adopt differentiated strategies. On one hand, increased financial investment and information empowerment may be directed toward village clinics—the safety net of rural healthcare—to consolidate localized gains in per capita visit volume, while also taking into account the functional positioning of community health stations. On the other hand, given the high share of chronic disease spending and the long-standing tendency to prioritize treatment over prevention, CCMC construction may gradually explore ways to move beyond a model that relies primarily on end-stage care, incorporating integrated medical-preventive services, health management, and disease prevention into performance evaluation, and encouraging large hospitals and grassroots institutions to jointly address source-based prevention, so as to promote the long-term sustainability of the healthcare system.

## Data Availability

The original contributions presented in the study are included in the article/supplementary material, further inquiries can be directed to the corresponding author.

## Footnotes

1 To avoid mathematically undefined values when taking logarithms in subsequent steps, we apply a small-shift correction: any standardized value ${x_{\mathrm{ij}}}^{\prime }=0$ is replaced with $10^{-8}$ .
